# High-level expression of a recombinant active microbial transglutaminase in *Escherichia coli*

**DOI:** 10.1186/s12896-015-0202-4

**Published:** 2015-09-15

**Authors:** Barbara Salis, Gaia Spinetti, Silvia Scaramuzza, Michele Bossi, Gloria Saccani Jotti, Giancarlo Tonon, Davide Crobu, Rodolfo Schrepfer

**Affiliations:** Bio-Ker S.r.l., Sardinia Scientific and Technological Park, Building 3, 09010 Pula, Cagliari Italy; IRCCS MultiMedica, Milan, Italy; Department of Biomedical, Biotechnological and Traslational Science (S.Bi.Bi.T.), University of Parma, Via Volturno 39, 43121 Parma, Italy

## Abstract

**Background:**

Bacterial transglutaminases are increasingly required as industrial reagents for *in vitro* modification of proteins in different fields such as in food processing as well as for enzymatic site-specific covalent conjugation of therapeutic proteins to polyethylene glycol to get derivatives with improved clinical performances.

In this work we studied the production in *Escherichia coli* of a recombinant transglutaminase from *Streptomyces mobaraensis* (microbial transglutaminase or MTGase) as enzymatically active chimeric forms using different expression systems under the control of both lac promoter or thermoinducible phage lambda promoter.

**Results:**

Thermoinducible and constitutive expression vectors were constructed expressing Met-MTGase with chimeric LacZ_1-8_PNP_1–20_ or LacZ_1–8_ fusion protein under different promoters. After transformed in competent *Escherichia coli* K12 strains were fermented in batch and fed-bach mode in different mediums in order to select the best conditions of expression.

The two most performing fusion protein systems namely short thermoinducible LacZ_1–8_Met-MTGase from NP668/1 and long constitutive LacZ_1–8_PNP_1–20_Met-MTGase from NP650/1 has been chosen to compare both efficiency of expression and biochemical qualities of the product. Proteins were extracted, purified to homogeneity and verified as a single peak obtained in RP-HPLC. The LacZ_1–8_PNP_1–20_Met-MTGase fusion protein purified from NP650/1 exhibited an activity of 15 U/mg compared to 24 U/mg for the shorter fusion protein purified from NP668/1 cell strain.

**Conclusions:**

Combining the experimental data on expression levels and specific activities of purified MTGase fusion proteins, the chimeric LacZ_1–8_Met-MTGase, which displays an enzymatic activity comparable to the wild-type enzyme, was selected as a candidate for producing microbial transglutaminase for industrial applications.

## Background

Transglutaminases (TGases, protein-glutamine γ − glutamyltransferase, E.C.2.3.2.13) are a large family of multifunctional enzymes occurring in several organisms, including mammals, invertebrates, plants and microorganisms. These enzymes catalyze an acyl transfer reaction between the γ-carboxyamide group of a peptide-bound glutaminyl residue (acting as acyl donor) and a variety of primary amines (acting as acceptor) including the ε-amino group of a peptide-bound lysine, resulting in the formation of a new γ-glutaminyl covalent link and ammonia [1].

It is widely demonstrated that transglutaminase is involved in several physiological process. For instance, it serves a key factor in pancreatic fl-cell during glucose-stimulated insulin release through Ca^2+^ dependent enzyme reaction in isolated islets of Langerhans [2].

Moreover, transglutaminase family is widely expressed in cardiovascular cells and in macrophages, and the recent studies have documented diverse roles for transglutaminases in cardiovascular pathophysiology, in chronic as well as the acute manifestations of atherosclerosis (e.g., plaque rupture). Transglutaminases can modulate several cardiovascular risk factors, especially hypertension [3].

The ability to promote covalent modifications of proteins by intra- and intermolecular linkages catalyzed by tranglutaminases is also exploited in industrial food as an established procedure to improve the texture and the nutritional value of different foods [4]. More recently, based on mild operative conditions and the recognition of specific substrate sequences, transglutaminases have been proposed as efficient catalysts for site-specific labeling of proteins with ligand molecules, as for example in the case of enzymatic pegylation of recombinant therapeutic proteins whose pharmacokinetics and/or pharmacodynamic properties can be modulated by covalent binding of high molecular weight poly(ethylene glycol) moieties [5].

Known microbial transglutaminases (MTGase) differ from the mammalian enzymes in that they lack any sequence homology, display Ca^2+^ independent activity and have smaller molecular masses [6]. Due to their properties, including Ca^2+^ independent activity, high reaction and broad specificity for acyl donors, microbial transglutaminases are advantageously used in industrial application both in food and in pharmaceutical fields.

A microbial transglutaminase (MTGase) from *Streptoverticillium mobaraensis (S.mobarensis)* has been isolated, characterized and cloned [7]. This protein, which consists of 331 amino acids with a molecular mass of 37.9 kDa, is biosynthesized as a pre-pro-protein, then secreted from cytoplasmic compartment as inactive pro-MTGase which is finally activated by proteolytic processing to the enzymatically active mature form [8].

MTGase was first prepared by conventional cultivation of wild-type strain of *S.mobaraensis* where the secreted inactive pro-enzyme was activated by endogenous proteases [9]. Later on, a number of reports have described the production of MTGase in different recombinant bacterial hosts such as *Streptomyces lividans* [10, 11]*, Lactococcus lactis* [12] and *Escherichia coli* [13]. Because of its protein cross linking activity, MTGase is a potentially toxic agent for the recombinant expression hosts if produced intracellularly in an active form. Therefore, the expression as functionally inactive pro-enzyme with subsequent *in vitro* activation by cleavage of the pro-sequence or, alternatively, the expression of mature MTGase as insoluble inclusion bodies followed by *in vitro* refolding is necessary.

In a previous work, we found that recombinant proteins bearing an N-terminal extension consisting of a few LacZ residues followed by the first twenty residues of the enzyme purine nucleoside phosphorylase (PNP_1–20_) when expressed in *Escherichia.coli (E.coli),* were invariably accumulated at high level inside the cell as cytoplasmic inclusion bodies [14]. In the present paper we describe the application of the same fusion protein technology for producing recombinant MTGase in *E.coli* according to different expression systems comprising a constitutive and thermoinducibile one under the control of the λ phage promoter. The results confirmed the critical importance of the N-terminal extension to obtain both high level expression of MTGase and its cytoplasmic segregation in inclusion bodies. Starting from washed inclusion bodies we also developed an efficient refolding and purification protocol to obtain a final MTGase preparation which displayed highly specific activity.

In conclusion, the procedure described in this work could potentially be applied for producing pure recombinant MTGase on a commercial scale for subsequent use as a convenient biocatalyst for the preparation of new protein conjugates.

To date, the mainly bacterial expression system to biosynthesise transglutaminases is *(S.mobarensis)*, this system however has some drawbacks, involving, e.g. problems related to post-translational protein modification [4]. In this work we present a cheaper and more efficient system based on the *E.coli* peculiarity to highly express recombinant proteins by inclusion body identifying and developing the most performing expression system, the procedure described could therefore potentially be applied for producing pure recombinant MTGase on a commercial scale for subsequent use as a convenient biocatalyst for the preparation of new protein conjugates.

## Methods

### General methods

All DNA manipulations, including restriction digestions, ligations and electrophoresis on agarose gels, were carried out as described [15]. Restriction and DNA-modifying enzymes were purchased from New England Biolabs (Beverly, MA, USA) and used according to the manufacturer’s instructions. PCR experiments were performed using a PCR thermal cycler (Gene Amp® PCR System 2700, Applied Biosystems), a high fidelity PCR system (PfuTurbo Hot Start and Easy A Hi Fi from Stratagene) and oligonucleotides synthesized by M-Medical (Milan, Italy). Plasmid extractions from agarose gels and PCR purifications were performed using Qiagen kits. The competent cells were transformed using the Bio-Rad *E. coli* Pulser transformation apparatus and selected using antibiotic resistance.

Soytone, yeast extract and all other chemicals and components for the preparation of culture media were from Merck, Sigma-Aldrich or Fluka.

### Synthetic cDNA coding for Met-MTGase

cDNA coding for mature *S.mobaraensis* MTGase (GenBank database accession n° DQ132977) optimized according to *E.coli* codon usage was synthesized by Sloning Bio Technology GmbH (Puchheim, Germany) using a proprietary genetic algorithm. Essentially, rare codons were replaced with the most frequently used *E.coli* codon for each particular amino acid. Other parameters such as GC-content bias, stability of the RNA secondary structure and length of direct and inverted repeats were also assessed following codon optimisation.

A starting ATG codon for Met residue at the N-terminus and two consecutive stop codons TAA TGA at C-terminus have been added. The synthetic gene was transferred to the *HpaI-XhoI* sites of plasmid pPCRScript (from Sloning, Puchheim, Germany) to give the vector PL444 enconding for Met-MTGase.

### Construction of thermoinducible vectors

The thermoinducible expression vectors were constructed starting from plasmid PL420 previously prepared in our laboratory for expressing interferon beta (IFN-β-1b) as chimeric LacZ_1–8_PNP_1–20_ fusion protein under the λ phage promoter, and characterized for the presence of the following main components:

- a 1375 bp fragment comprising the P**R** and P**L** promoters and the sequence coding for the thermosensitive repressor cI857 isolated from plasmid pND201 [16]
- a coding sequence for IFN-β-1b immediately dowstream of a oligonucleotide sequence coding for the first 8 residues of LacZ protein (UniProt/SwissProt Accession No. Q37953) and the first 20 aminoacids of *E.coli* purine nucleoside phosphorylase [14]
- the kanamycine resistance gene and the pUC19 origin of replication
- a multicloning site.

In vectors containing P**R** and P**L** promoters of bacteriophage λ, transcription may be controlled by the λ repressor, supplied by expression of *cI* gene. If a temperature-sensitive allele of *cI* is used (e.g., cI857), transcription from the strong promoters is repressed in cells growing at 30 °C. At 42 °C, the thermolabile repressor is inactivated and transcription is enabled [16]. In our experiments we noticed that the strong promoter is repressed until 37 °C, therefore we used this temperature for cell growing and 42 °C for expression of protein.

**Fig. 1:**
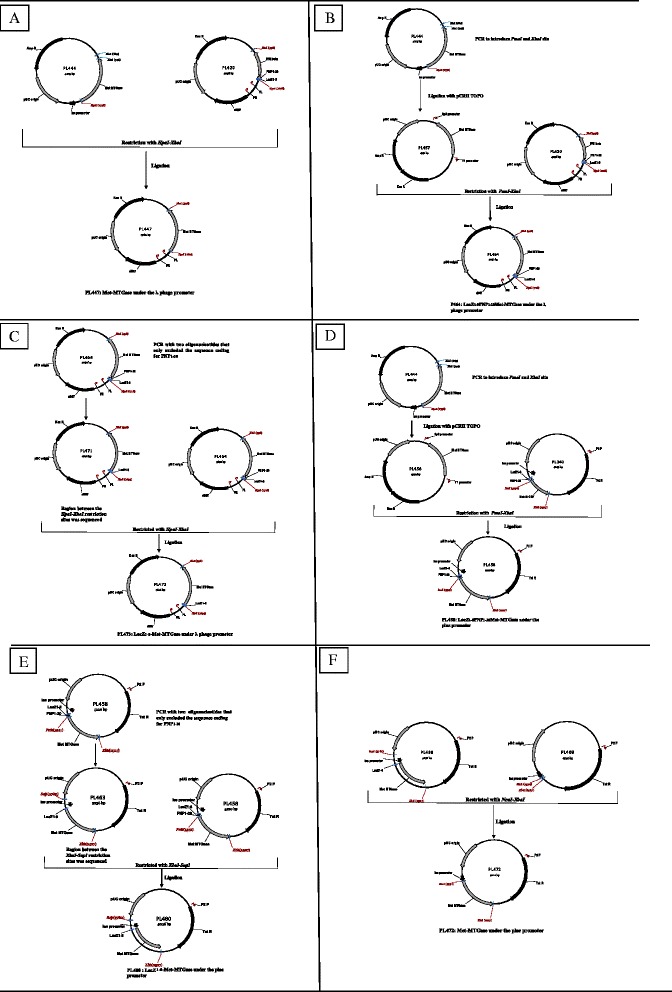
Construction and structure of MTG expression plasmids. Thermoinducible plasmids, panel: **a**, **b** and **c** (PL447, PL464 and PL473) and constitutive plasmids, panel: **d**, **e** and **f** (PL458, PL480 and PL472). The restriction enzyme sites used for construction of the plasmids are indicated. Additional details are described in Methods

The sequence coding for Met-MTGase_1–332_ was excised by *HpaI-XhoI* digestion of PL444 and cloned in the same restriction sites of PL420 to obtain the vector PL447 for the expression of mature Met-MTGase enzyme under the λ phage promoter (Fig. 1, panel a).

The sequence coding for Met-MTGase_1–332_ was PCR amplified from PL455 using the sense primer **5′***GTTTAAAC*GT*ATG*GACAGCGATGACCG **3′** (with the *PmeI* site and the Met-MTGase start codon underlined) and the antisense primer **5′***CTCGAGTCATTA*TGGCCAGCCTTGTTTAAC **3′** (with the *XhoI* site and the complementary sequence of a double stop codon underlined).

The amplified fragment was cloned in pCRII TOPO (TA Cloning kit, Cat n° K2020-20, Invitrogen) obtaining PL457 and later restricted by *HpaI-XhoI* and cloned in the same restriction sites of PL420 dowstream of sequence coding for LacZ_1–8_PNP_1–20_ to obtain the vector PL464 for the expression of the chimeric protein LacZ_1–8_PNP_1–20_-Met-MTGase under the λ phage promoter (Fig. 1, panel b).

The vector for expressing LacZ_1–8-_Met-MTGase was constructed using an intermediate vector. The plasmid, called PL471, was prepared by PCR amplification of PL464 with two oligonucleotides that only excluded the sequence coding for PNP_1–20_ using the sense primer **5′***ATG*GACAGCGATGACCGCGTG **3′** (where the Met-MTGase start codon is underlined) and the antisense primer

**5′***GGA*AGAATTCGTAATCATGGTCATAGGTTAAC **3′** (where the first underlined codon is the complementary codon for the last aminoacid of LacZ_1–8_ fragment). Since plasmid PL471 had been amplified by PCR, the region between the *HpaI-XhoI* restriction sites of PL471 was sequenced and then transferred into PL464 to obtain the final vector for expressing LacZ_1–8_Met-MTGase under the λ phage promoter denominated PL473 (Fig. 1, panel c).

### Construction of constitutive vectors

The constitutive expression vectors were constructed starting from plasmid PL340 previously prepared in our laboratory for expressing Met-G-CSF as hybrid LacZ_1–8_PNP_1–20_ fusion protein under the lac promoter, and characterized for the presence of the following main components:

- the lac promoter of commercially available pUC18 vector (ATCC N° 37253)
- a coding sequence for Met-G-CSF immediately dowstream of a oligonucleotide sequence coding for the first 8 residues of LacZ protein (UniProt/SwissProt Accession No. Q37953) and the first 20 aminoacids of *E.coli* purine nucleoside phosphorylase [14]
- the tetracycline resistance gene and the pUC18 origin of replication
- a multicloning site.

pUC18 derived vectors containing the inducible lac promoter when transformed into JM109 *E.coli* strains constitutively express the encoded genes without any addition of inducing agents such as IPTG or lactose [14, 17].

The sequence coding for Met-MTGase_1–332_ was amplified by PCR from PL444 using the sense primer **5′***GTTTAAAC*GT*ATG*GACAGCGATGACCG **3′** (with the *PmeI* site and the Met-MTGase start codon underlined) and the antisense primer **5′***TCTAGA*G*TCATTA*TGGCCAGCCTTGTTTAAC **3′** (with the *XbaI* site and the complementary sequence of a double stop codon underlined).

The amplified fragment was cloned in pCRII TOPO (TA Cloning kit, Cat n° K2020-20, Invitrogen) obtaining PL456 and later restricted by *PmeI-XbaI* and cloned in the same restriction sites of PL340 downstream of sequence coding for LacZ_1−_8PNP_1–20_ to obtain the vector PL458 for the constitutive expression of the chimeric protein LacZ_1–8_PNP_1–20_Met-MTGase (Fig. 1, panel d).

The vector for expressing LacZ_1–8_Met-MTGase under the lac promoter was constructed using an intermediate vector called PL463.

The vector was prepared by PCR amplification of PL458 with two oligonucleotides that only excluded the sequence coding for PNP_1–20_ using the sense primer **5′***ATG*GACAGCGATGACCGCGTG **3′** (with Met-MTGase start codon underlined) and the antisense primer **5**′ *GGA*AGAATTCGTAATCATGGTCATAGCTG 3′ (where the first underlined codon is the complementary codon for the last aminoacid of LacZ_1–8_ fragment). Since plasmid PL463 had been amplificated by PCR, the region between the *XbaI-SapI* restriction sites was sequenced and then transferred into PL458 restricted with the same enzymes to obtain the vector for the constitutive expression of chimeric LacZ_1–8_Met-MTGase denominated PL480 (Fig. 1, panel e).

In order to obtain a plasmid with lac promoter that expresses Met-MTGase was used a plasmid called PL469 previously prepared in our laboratory. PL469 contains the tetracycline resistance gene, the pUC18 origin of replication and the LacZ_1–8_ between *NcoI-XbaI* sites. So PL480 was restricted with *NcoI-XbaI* and the fragment was inserted into PL469 restricted with the same enzymes to obtain the vector for the constitutive expression of Met-MTGase denominated PL472 (Fig. 1, panel f).

### Cloning and expression of different Met-MTGase and chimeric Met-MTGase coding vectors

Competent *Escherichia coli* K12, JM109 cell strains were transformed by electroporation with several purified plasmids as described in Table 1.

**Table 1:** List of plasmids and related strains, engineered for different MTGase expression forms in *Escherichia coli*, fermented in shake flask for protein expression detection

Plasmid	Type	Cell strain	Promoter	Cloned gene	Antibiotic resistance
PL444 (Sloning)	Starting	NP647	Lac promoter	Met-MTGase_1–332_	Amp
PL447	Expression	NP637	λ-phage	Met-MTGase_1–322_	Kan
PL456	Intermediate	NP648	T7-SP6 Promoter	Met-MTGase_1–332_	Amp-Kan
PL457	Intermediate	NP649	T7-SP6 Promoter	Met-MTGase_1–332_	Amp-Kan
PL458	Expression	NP650	Lac promoter	LacZ_1–8_PNP_1–20_ Met-MTGase_1–332_	Tet
PL463	Intermediate	NP655	Lac promoter	LacZ_1–8_Met_−_MTGase_1–332_	Tet
PL464	Expression	NP656	Phage λ	LacZ_1–8_PNP_1–20_Met-MTGase_1–332_	Kan
PL469	Expression	NP663	Lac promoter	LacZ_1–8_	Tet
PL471	Intermediate	NP666	Phage λ	LacZ_1–8_Met-MTGase_1–332_	Kan
PL472	Expression	NP667	Lac promoter	Met-MTGase_1–332_	Tet
PL473	Expression	NP668	Phage λ	LacZ_1–8_Met-MTGase_1–332_	Kan
PL480	Expression	NP676	Lac promoter	LacZ_1–8_Met-MTGase_1–332_	Tet

Transformed cells were streaked onto agar plates prepared in LS_10_ medium 5 g/l yeast extract, 10 g/l soytone, 10 g/l sodium chloride containing 12.5 mg/l tetracycline and incubated in a static incubator (B 12, Heraeus Instruments) overnight at 37 °C. Four isolated colonies of each transformed strain were selected from the agar plates and used to inoculate 10 ml LS_10_ culture medium in a 100 ml shake flasks, which were incubated at 220 rpm and 37C overnight in an orbital shaker (Innova 4330, New Brunswick). About 6 ml of each of the incubated cell suspensions were adjusted to a final concentration of 16 % v/v glycerol, distributed in four cryo-vials and stored frozen at −80 °C.

About 100 μl were taken from each of four cryo-vials of the six recombinant strains and used to inoculate 10 ml of a rich, complex culture medium (18 g/l yeast extract, 10 g/l soy peptone, 10 g/l glycerol, 3.2 g/l K_2_HPO_4_, 0.6 g/l KH_2_PO_4_, 1 g/l MgSO_4_.7H_2_O and 0.03 g/l kanamycin) in 100 ml shake flasks incubated overnight using an orbital shaker at 220 rpm and 37 °C.

1.5 ml samples of each overnight cultures of constitutively expressed recombinant Met-MTGase (NP667, NP650 and NP676 strains) were pelleted by centrifugation at +4 °C, washed with 1.6 ml of 20 mM Tris–HCl—1 mM EDTA—pH 7 buffer, suspended in phosphate buffer saline to OD_600nm_ value of about 100 and analysed by reducing SDS-PAGE with Coomassie blue staining.

Overnight cultures of recombinant Met-MTGase expressed under the λ phage promoter (NP637, NP656 and NP668) were diluted with 50 ml of rich medium and incubated in 250 ml shake flasks at 37 °C for about 2 h up to an OD_600_ value of 0.5 ± 0.1. Afterwards the cultures were then incubated for a further 4 h in orbital shakers at 42 °C.

1.5 ml taken from of each cell suspensions were prepared and analysed by reducing SDS-PAGE as described.

Since no significant difference in terms of cell growth and recombinant protein expression was observed among the four clones examined, one from each recombinant strain was selected at random for Molecular Biology cell banks to be used for large scale fermentations.

### Large scale preparation of MTGase fusion proteins

The production of the MTGase fusion proteins has been performed using 10-litre scale stirred tank bioreactors (Biostat C, B. Braun). The compositions of the culture media employed were originally tested and verified for the production of other recombinant fusion proteins manufactured using the same cell host. Starting from a vial of the respective Molecular Biology cell banks, the process consisted of inoculum preparation in shake flask followed by a 10-litre scale batch or fed-batch production step.

Pre-inoculum was made starting from one vial of the Molecular Biology cell bank in culture medium FMYb, an animal free derived media composed by a pre defined solution of yeast extract, glycerol, vitamins and minerals with apposite antibiotic and then inoculated in 10 litres of FMYb culture medium for batch fermentation.

Furthermore, FM1 medium, a solution of yeast extract, glycerol, soy peptone, minerals and vitamins, was used in the same conditions for feed-batch and batch fermentation in order to investigate the best condition of expression.

### Bach and Fed-batch process conditions

One vial of the Molecular Biology cell bank was inoculated in 500 ml/flask of FMY and FM1 medium and cultivated 37 °C at 220 rpm in an orbital shaker (Innova 4300, New Brunswick). Afterwards 100 ml of pre-inoculum was inoculated in 10 litres culture medium: (1 % v/v) for FMY or (5 % v/v) for FM1.

Cultivation followed for 5 h at 37 °C with air flow 1 v/v/min, pH was controlled to 7.0 ± 0.1 using 12.5 % v/v NH_4_OH and 20 % v/v H_3_PO_4_. The pO_2_ saturation was 20 % controlled by stirring rate at range of 300–1300 rpm for FMY medium and 300–1500 rpm for FM1, with a mix of pure oxygen:air at 3:20 supplied upon reaching highest stirring rate. Minimum stirring speed was settled at 300 rpm for both media, direct air sparging was provided using ring sparger with holes on upper side, agitation using 3 separate flat-bladed (6 blades) Ruston impellers. Finally, foaming was controlled by antifoam addition (Antifoam 204, mixture of organic polyether dispersions).

On-line data acquisition included pH, stirring rate, pO_2_, temperature and air flow rate, off-line analyses included measuring the OD_600_ and the pH during fermentation.

For fed-batch condition 8 litres of sterile culture media FM1 was prepared in bioreactor (Biostat C). In correspondence with sharp pO_2_ increase, indicating slower growth rate, the feed profile was 2 litres exponential feeding, 8-hour duplication time, 5 replications with air flow 1 v/v/min. Foaming and pO_2_ was controlled as reported for batch fermentation.

### Purification methods

Upon completing fermentation, the cell broths were harvested and centrifuged (equivalent: 9.000 RCF; 15 min; +4 °C) to obtain concentrated cell paste, which was then stored frozen at −20 °C until required.

The LacZ_1–8_Met-MTGase fusion proteins, expressed in the form of cytoplasmic inclusion bodies, were recovered by suspending the cell paste (10 % w/v) in 50 mM sodium phosphate buffer, pH 7.4 containing 100 m M NaCl, 5 mM EDTA and 0.1 % v/v Triton X100 at 4 °C and passing, for three successive cycles, through a high-pressure homogenizer (Manton-Gaulin) operated at 650 ± 25 bar.

The cell homogenate (about 6 l) was then cooled to below +10 °C and batch centrifuged (equivalent: 9.000 RCF; 15 min; +4 °C) to pellet the inclusion bodies. The inclusion bodies were then washed twice by suspension at +4 °C in 4 l of 50 mM sodium phosphate buffer, pH 7.4 containing 100 m M NaCl, 5 mM EDTA, under agitation for 30–60 min and then collected by centrifugation.

Washed inclusion bodies were finally suspended 1:1 in 50 mM Tris–HCl, 5 mM EDTA, pH 8.0 to form an homogeneous slurry in a volume of about 3–4 % of initial cell broth volume, suitably aliquoted and stored frozen at −20 °C until required.

LacZ_1–8_Met-MTGase inclusion bodies were dissolved (10 % w/v) in the solubilisation buffer (8 M urea containing 50 mM sodium acetate and 20 mM dithiothreitol (DTT), pH 5.2) and incubated, in mild agitation, for 1 h at room temperature. The solubilisation buffer was added to the washed inclusion bodies preparation at a constant flow rate within about 10 ml/min, to obtain a protein concentration of about 3 mg/ml.

The solubilised LacZ_1–8_Met-MTGase solution was then diluted 20-fold to a concentration of 0.32 M Urea using 20 mM sodium acetate, pH 5.2. After 1 h of incubation in mild agitation at +4 °C, the pH was shifted from 5.2 to 6.0 by adding 1 M NaOH.

The refolded LacZ_1–8_Met-MTGase protein solutions were clarified by centrifugation at 17.700 RCF for 10 min and at  4° to remove any precipitated material and concentrated using a 10 kDa cut-off ultrafiltration membrane (PrepScale-1 ft^2^, Millipore). The solubilization and refold reagents used were then removed by dialysis against 50 mM Tris acetate, pH 8.00 and the protein concentrate was drained down so that the equipment rinsed with the dialysis buffer recover residual product.

**Fig. 2:**
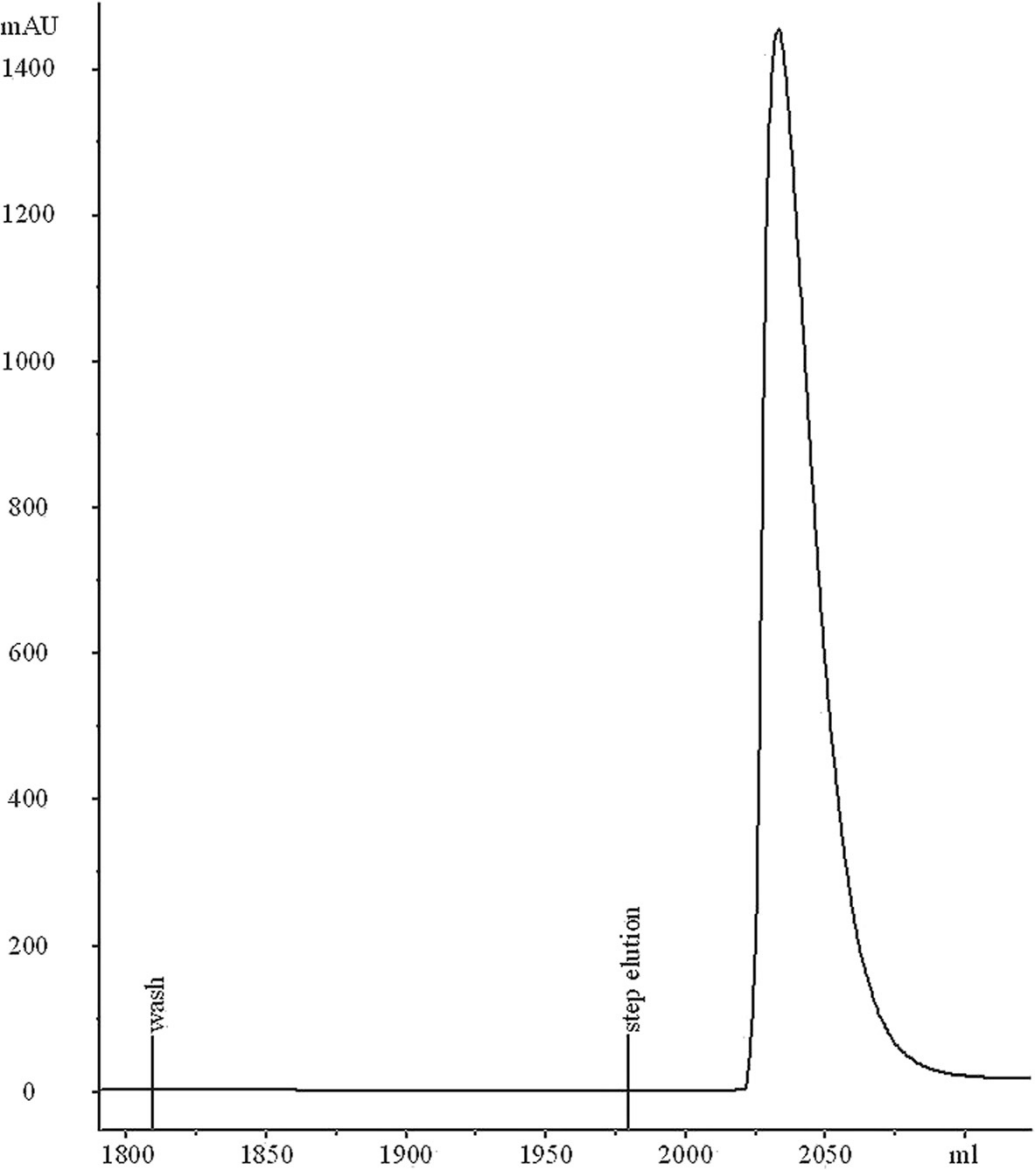
Hydrophobic interaction chromatography of LacZ_1–8_Met-MTGase on phenyl Sepharose with a step elution of 0,5 M sodium chloride, 50 mM sodium acetate, pH 5.5 buffer, at room temperature

LacZ_1–8_Met-MTGase was purified by hydrophobic interaction chromatography on Phenyl Sepharose 6 Fast Flow (high substitution) (Fig. 2). The resin was packed to a column height of 22 cm, column diameter of 16 mm and the column operated at linear flow rates up to 300 cm/h. The column was equilibrated with 3.5 M sodium chloride, 50 mM sodium acetate at pH = 5.5 (acetic acid). The load material consisted of the dialysed sample adjusted to 3.5 M NaCl, acidified to pH 5.5 using acetic acid diluted 1:20 and filtered 0.22 μm. After loading, the column was washed (4 CV) using the equilibration buffer. LacZ_1–8_Met-MTGase was eluted using a step elution with 0.5 M sodium chloride 50 mM sodium acetate, pH 5.5. A buffer exchange column was necessary to put the material in the right conditions for the enzyme-catalysed reaction of pegylation.

### Analytical methods

Expression of MTGase and MTGase fusion proteins was monitored by staining with Coomassie Blue R-250 after 12 % SDS-PAGE analysis [18] of solubilized cells using a Bio-Rad mini-gel apparatus as well as by RP-HPLC carried out on a C4 Vydac 214TP52 column (2.1 × 250 mm, 5 μm particle size, Grace) at 40 °C with UV detection at 215 nm. MTGase was eluted at 0.2 mL/min starting from the mobile phases A (0.1 % v/v TFA in H2O) and B (0.08 % v/v TFA in CH3CN) with the following linear gradients: from 30 % to 59 % of phase B in 13 min and from 59 % to 85 % of phase B in 7 min.

Transglutaminase activity was measured by a described colorimetric method based on hydroxamate formation [19]. Briefly, 0.2 mL of 200 mM Tris-buffer pH 6.0 containing 36 mM carbobenzoxy-glutaminyl-glycine, 100 mM hydroxylamine and 10 mM reduced glutathione were incubated for 10 min at 37 °C with 30 μL of test sample. The enzymatic reaction was stopped by adding 0.5 mL of 12 % trichloroacetic acid. After that, 0.5 mL of 5 % ferric chloride exahydrate solutions were added and the resulting red color was measured at 525 nm. A calibration curve was prepared using commercially available L-glutamic acid γ-monohydroxamate. One unit of MTGase activity is defined as the amount of enzyme that catalyzes the formation of 1.0 μmole of hydroxamate/min at the above reported conditions. Specific activity is expressed as MTGase units/mg of protein.

Purity and concentration of MTGase were determined by HPLC analysis. SE-HPLC was carried out using a TSKgel SuperSW3000column (4.6 × 300 mm, 4 μm particle size, Tosoh Bioscience). The column was thermostated at 25 °C and eluted with 0.05 M NaH_2_PO_4_, 0.4 M NaClO_4_, pH 6.0, at a flow rate of 0.35 mL/min. The UV detector was set at 215 nm. RP-HPLC was performed on a C4 Vydac 214TP54 column (4.6 × 250 mm, 5 μm particle size, Grace), thermostated at 55 °C and equilibrated with 75 % (v/v) buffer A (0.1 % TFA v/v in H_2_O) and 25 % (v/v) buffer B (0.08 % TFA v/v in CH_3_CN). The enzyme was eluted at a flow rate of 0.8 mL/min with a linear gradient from 25 % to 45 % of buffer B in 23 min, recording the absorbance at 215 nm. Quantization was carried out by the external standard method, using a standard bovine serum albumin (BSA) solution as reference solution.

We also verified the functionality of the obtained recombinant transglutaminase by the process for site-specific pegylation of Met-GCSF developed by Bio-Ker [20]. Briefly Met-GCSF was submitted to covalent conjugation at glutamine 135 residue with a 20 kDa amine derivative of polyethylene glycol (PEG_20kDa_-NH_2_) via enzyme-catalysed reaction using the purified Ajinomoto “wild type” microbial transglutaminase in comparison with the purified LacZ_1–8_Met-MTGase from NP668/1. The experimental conditions where PEG/Met-GCSF ratio 10:1, [Met-GCSF]_final_ 2 mg/ml, [TGase]_final_ 0.25 U/ml solution, 4 °C, buffer KH_2_PO4 20 mM and pH 8,1.

## Results and discussion

**Fig. 3:**
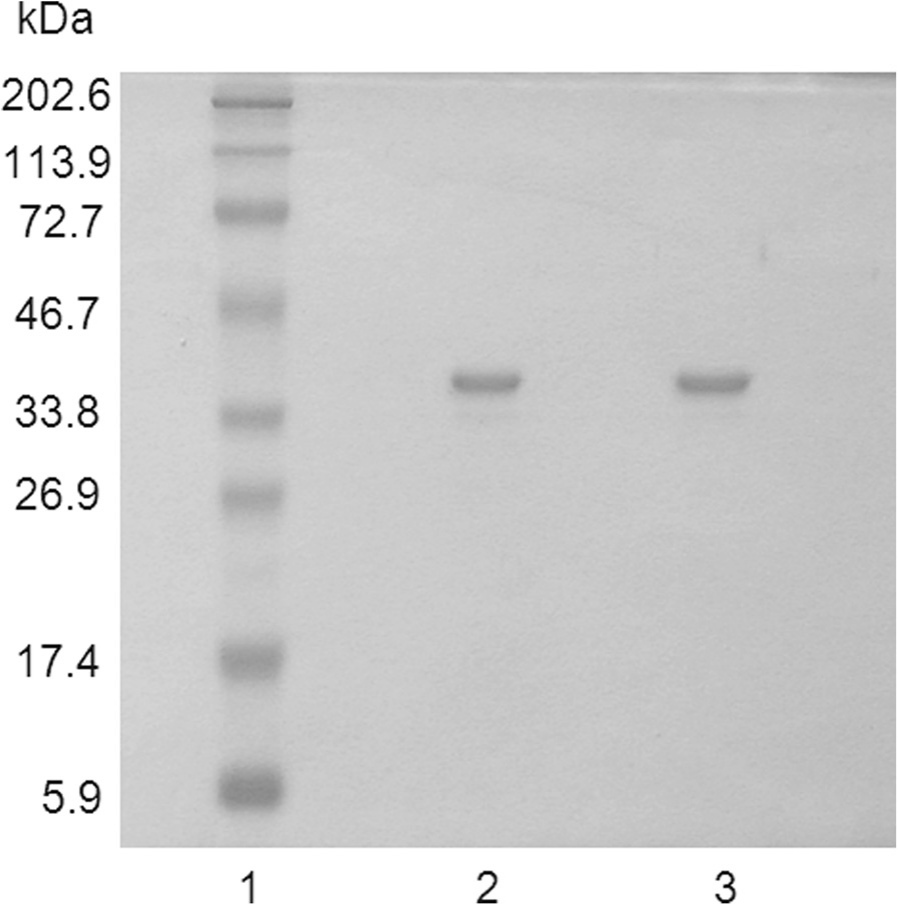
SDS-PAGE electrophoresis of reduced and non reduced MTGase samples stained by Coomassie Blue R-250. Lane 1, protein markers; lane 2, MTGase in non reducing sample buffer; lane 3, MTGase in reducing sample buffer

**Fig. 4:**
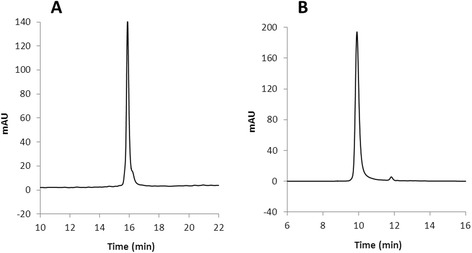
Reverse-phase (**a**) and size-exclusion HPLC (**b**) analysis of purified MTGase from NP668

Purity of LacZ_1–8_Met-MTGase has been evaluated by electrophoresis and HPLC analysis. A single protein band was observed on SDS-PAGE gels under both reducing and non reducing conditions at the expected molecular weight (Fig. 3). The size-exclusion and reverse-phase chromatograms showed a single symmetric peak corresponding to 99.6 % and 88.8 % protein purity, respectively, under native conditions (Fig. 4). The enzyme purity was confirmed by TGase activity assay which gave a specific activity of 24U/mg.

### Expression systems and assessment

Four different recombinant cell strains, expressing a Met-MTGase fusion protein with two different N-terminal fusion partners in two separate expression systems, have been assessed. Specifically, the long LacZ_1–8_PNP_20_Met-MTGase fusion protein was expressed in cell strains NP650/1 under constitutive lac promoter and NP656/1 under the thermoinducible phage lambda promoter.

The shorter fusion protein LacZ_1–8_Met-MTGase, having just the first eight N-terminal amino acids of beta galactosidase at the N-terminal, was expressed in thermoinducible cell strain NP668/1 and constitutive cell strain NP676/1 (later replaced by NP676/2). None appreciable level of protein expression could be discerned by SDS-PAGE analysis for the strains expressing Met-MTGase without an N-terminal fusion partner (NP637/1 and NP667/1). All the others strains possessing an N-terminal fusion partner yielded a good level of protein expression. Apart from the NP676 cell strain, expressing LacZ_1–8_Met-MTGase, which was characterised by a long time of growth, the other three cell strains were incubated without problems suggesting that expression of Met-MTGase protein was not deleterious for the cell host.

### Fermentation studies

As a result of preliminary experiments, a total of four different cell strains were selected to be tested using the 10-litre fermentation process, namely NP650/1, NP656/1, NP668/1 and NP676/1 (Table 2). As described below the NP676 cell strain was transformed twice (NP676/2) and also fermented.

**Table 2:** Fermentation of four most performing strains expressing MTGase, comparison of two expression systems

Fusion protein	Batch #	System	FB/batch	Final OD_600_	MTGase (g/batch)	Wet mass (g)
LacZ_1–8_ Met-MTGase	NP676/C002	Constitutive	Batch	60	26,7	693
	NP676/C001		Fed-batch	57	24,1	709
	NP668/009	Inducible	Batch	54	25,0	540
LacZ_1–8_PNP_1–8_ Met-MTGase	NP650/C005	Constitutive	Batch	58	16,5	790
	NP650/C006		Fed-batch	74	62,0	748
	NP656/C009	Inducible	Batch	46	39,2	335
	NP656/C010		Fed-batch	56	42,7	490

The constitutive NP650/1 cell strain yielded a very high volumetric productivity in terms of cell broth volume of more than 6 g/l LacZ_1–8_PNP_1–20_Met-MTGase fusion protein for a fed-batch fermentation process. Since this fed batch process typically lasted from 30 to 45 h, depending upon the adopted feeding profile, the same cell strain was tested using a batch process shortening the production time to about 8 h. However, despite a comparable final biomass content, a relatively low volumetric productivity of only 1.65 g/l was obtained. These results were confirmed by repeating the processes. In any case, cytoplasmic expression of the LacZ_1–8_PNP_1–20_Met-MTGase fusion protein didn’t appear to have any appreciable toxic effect or to be detrimental to the growth of the host cell strain.

The same constitutive expression system was tested for Met-MTGase having the shorter N-terminal fusion partner (cell strain NP676/1) but all of four fed-batch production runs were terminated due to excessive foaming.

Considering that in other similar experiments, constitutive systems expressing long LacZ_1–8_PNP_1–20_Met-MTGase fusion protein had no apparent toxic effect on the cells, we repeated the previous experiment transforming again the plasmid in a newly aliquot of competent cells obtaining a new cell bank named NP676/2. The new transformed cell strain was also characterized by slow growth, anyway two fed-batch fermentation processes have been completed, resulting in a final volumetric productivity of about 2.5 g/l and a specific productivity, product titre in terms of grams cell wet weight, of about 36 mg/g.

Five fermentation batches using the thermo-inducible NP656/1 cell strain expressing the LacZ_1–8_PNP_20_Met-MTGase fusion protein were performed, the fermenter temperature was shifted to 42 °C at an OD_600_ value of 13 after 3.5 h incubation and a final product yield of 3 g/l was obtained. In another batch the temperature shift was performed after 5.5 h incubation, at an OD_600_ value of 36, resulting in a lower final volumetric productivity of 1.65 g/l.

In both batches, an high level of protein expression has been obtained after only four hours post-induction and no expressed product were observed before induction. In a third batch, the temperature shift was performed after 2 h incubation at a very low OD_600_ value of 4.5 which resulted in a low final biomass content (final OD_600_ value of 18) and a lower product yield of 1.3 g/l. The highest volumetric productivity, 3.85 g/l, was obtained when the temperature shift was performed at an OD_600_ value of 18, corresponding to the mid-point of the exponential growth phase.

In addition to the above described fermentations operated in a batch mode, similar five batches were run in a fed-batch mode. As result, final biomass and concentrations were comparable, but high expression yield of up to 4 g/l were obtained. Considering only the fed-batch processes performed using the optimal conditions, a mean specific productivity value of about 80 mg/g was obtained for NP650/1 compared to about 100 mg/g for NP656/1.

The thermoinducible NP668/1 cell strain expressing the shorter LacZ_1–8_MTGase fusion protein yielded a maximum volumetric productivity of about 3 g/l.

This cell strain was tested according to the batch production mode only and, the optimal point for the temperature shift to 42 °C corresponded to an OD_600_ value of about 20 (Table 3) giving a mean specific productivity of 40 mg/g cell wet weight, while in the same conditions were obtained for NP656/1 a productivity of 100 mg/g. Shifting the temperature at an OD_600_ value of 30 or more resulted in a markedly lower yield of 1.2–2.2 g/l, whereas a value of 2.1 g/l was obtained at a low OD_600_ value of 13.

**Table 3:** List of NP668/1 fermentation batches to investigate the optimal point of temperature shift

Batch #	OD_600_	LacZ_1–8_Met-MTGase (mg/l)	Wet mass (g)	LacZ_1–8_Met-MTGase (mg/g)
NP668/C001	58	1117	744	15,0
NP668/C002	64	2191	604	36,3
NP668/C003	56	3085	493	62,6
NP668/C004	51	1548	646	24,0
NP668/C005	58	2907	590	49,3
NP668/C006	60	2376	580	41,0
NP668/C007	58	2761	560	49,3
NP668/C008	42	2109	384	54,9
NP668/C009	54	2500	540	46,3
NP668/C010	53	2520	538	46,7
NP668/C013	60	2633	591	44,6
NP668/C014	59	2196	564	38,9
NP668/C015	60	1939	572	33,9
NP668/C016	50	2157	575	37,5
NP668/C017	37	1377	522	26,4
NP668/C018	33	2415	497	48,6
NP668/C019	32	1677	571	29,4
*Mean*	*52*	*2206*	*563*	*40,3*
*Sd*	*10*	*541*	*74*	*12,0*

The constitutive NP650/1 cell strain gave the highest volumetric productivity of 6.15 g/l, on the other hand, the thermoinducible cell strain NP656/1 exhibited a lower protein yield, but showed a more specific productivity in terms of mg of protein/cells (wet weight).

For both systems was evident that long LacZ_1–8_PNP_1–20_Met-MTGase fusion protein yielded both a higher volumetric and specific productivity, demonstrating the advantage of co-expressing the MTGase protein with the PNP_1–20_ peptide. It should be pointed out that this benefit is somewhat reduced if the additional mass, corresponding to about 10 % of the expressed protein, of the PNP_1–20_ peptide itself is taken into account.

### Purification and quality assessment

According to the preliminary fermentation process studies, the two most performing fusion protein systems namely short thermoinducible LacZ_1–8_Met-MTGase (NP668/1) and long constitutive LacZ_1–8_PNP_1–20_Met-MTGase (NP650/1) has been chosen to compare both efficiency of expression and biochemical qualities of the product.

Proteins were extracted and purified to homogeneity and so verified as a single peak obtained in RP-HPLC. The LacZ_1–8_PNP_1–20_Met-MTGase fusion protein purified from NP650/1 exhibited an activity of 15 U/mg compared to 24U/mg for the shorter fusion protein purified from NP668/1 cell strain (Table 4). The lower activity obtained for the LacZ_1–8_PNP_1–20_Met-MTGase could be attributable to the presence of the long PNP_1–20_ peptide chain, therefore, despite the lower protein expression yield obtained, the thermoinducible cell strain NP668/1 expression has been chosen as most performing expression system.

**Table 4:** Enzymatic activity of purified MTGase, comparison among the two best performing expression systems and wild type

NP650C001	NP668C003	AJ021
LacZ_1–8_PNP_1–20_Met-MTGase	LacZ_1–8_Met-MTGase	Wild type
15,1 U/mg	24 U/mg	22 U/mg

**Fig. 5:**
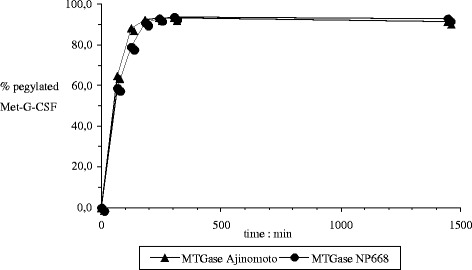
Kinetic profile functionality of NP668 versus Ajinomoto MTGase by comparing the enzymatic activities in covalent conjugation of 20 kDa amine derivative of polyethylene glycol at glutamine 135 residue of Met-G-CSF

Comparison of the functionality of the LacZ_1–8_Met-MTGase fusion protein (NP668/1) versus the Ajinomoto “wild type” microbial transglutaminase showed a same reaction kinetic profile for both products. In both cases the reaction reached the plateau after four hours and the reaction yields were comparable likewise the chromatographic profiles (SE-HPLC) (Fig. 5).

## Conclusions

Due to the implication of MTGase in many industrial and pharmaceutical fields, the interest in finding the better expression conditions of this enzyme is broadly widespread [21].

In this work we have described a series of studies aimed to determine the better strategy to express MTGase comparing constitutive versus thermoinducible expression systems rather than expressing the protein by fusion protein or wild type. Moreover a comparison between two fusion partner (LacZ_1–8_PNP_1–20_ and LacZ_1–8_) has been done. We showed that the expression of a MTGase fusion protein is advantageous in terms of fermentation yield and that the use of the short LacZ_1–8_Met-MTGase fusion protein improves the enzymatic activity of the product obtaining an activity value comparable to that of the “wild type” transglutamiase (Table 4).

Thereby, the importance to have a reliable protein activity expression combined with a fast and robust method of purification led to the choice of NP668/1 as reference strain to produce Met-MTGase.
